# The ATCC genome portal: 3,938 authenticated microbial reference genomes

**DOI:** 10.1128/mra.01045-23

**Published:** 2024-01-30

**Authors:** Scott V. Nguyen, Nikhita P. Puthuveetil, Joseph R. Petrone, Jade L. Kirkland, Kaitlyn Gaffney, Corina L. Tabron, Noah Wax, James Duncan, Stephen King, Robert Marlow, Amy L. Reese, David A. Yarmosh, Hannah H. McConnell, Ana S. Fernandes, John Bagnoli, Briana Benton, Jonathan L. Jacobs

**Affiliations:** 1 ATCC, University Blvd, Manassas, Virginia, USA; Indiana University, Bloomington, Indiana, USA

**Keywords:** genomics, database, quality assurance, bioinformatics, computational biology, microbial genomics, virology, mycology, bacteriology, protistology

## Abstract

The ATCC Genome Portal (AGP, https://genomes.atcc.org/) is a database of authenticated genomes for bacteria, fungi, protists, and viruses held in ATCC’s biorepository. It now includes 3,938 assemblies (253% increase) produced under ISO 9000 by ATCC. Here, we present new features and content added to the AGP for the research community.

## ANNOUNCEMENT

The ATCC Genome Portal (AGP) is a resource for genomic data provenance and traceability for the scientific community (1, 2). While whole-genome sequencing data for many ATCC strains have been published for decades, the associated genome assemblies are not necessarily directly traceable to the culture collection. Existing public-domain genome sequences may be derived from domesticated laboratory strains, cultures shared between laboratories, or materials that lack a proper chain of custody from the originating source. Genetic drift accumulated by passaging microbial strains has been demonstrated to exert a significant influence on phenotypes observed between isolates of the same strain (3–5). This variation can potentially lead to erroneous conclusions regarding the characteristics of strains under investigation (6, 7). By contrast, the reference genomes in the AGP are generated in-house directly from nucleic acids extracted from the collection’s authenticated materials and they are sequenced and assembled using genomics and bioinformatics workflows developed by ATCC (2, 3). Furthermore, data for the AGP are produced using ISO 9000—compliant procedures and workflows, further improving the quality and traceability of the resulting assemblies (3, 8). Authenticated reference genomes within the AGP can provide the research community insight into the genetic stability and pedigree of strains sourced from the collection (9) and avoid pitfalls associated with the “reproducibility crisis” (10). Standardized metadata also play a critical role in the usability and reproducibility of genomic data (11, 12).

The AGP provides a valuable resource for type strain genome assemblies, especially for species without reference genomes in NCBI. Currently, 1,057 of the assemblies in the AGP are for microbial type strains, 755 are for microbes never previously subjected to whole-genome sequencing, and 18 are for microbes included in NCBI’s list of high-priority prokaryotic species which have no existing genome assemblies (13). In addition, notable prokaryotic species with assemblies in the AGP, but not found in NCBI databases, include *Tatumella terrea*, *Pseudodesulfovibrio halophilus*, *Neptunomonas naphthovorans*, and *Halanaerobium lacusrosei*. At the time of this writing, the AGP also includes genomes for 264 fungi, 308 viruses, and 4 protists. Examples of fungal genomes in the AGP that represent taxa not present in NCBI include *Hyphozyma lignicola*, *Hyphozyma variabilis*, *Filobasidium magnum*, *Dactylella leptospora,* and *Arcopilus cupreus*.

The AGP is the first authenticated and fully traceable microbial genome reference database that provides end-to-end data provenance from biological materials to data. New genome assemblies are released to the AGP monthly. Access channels for the data include downloads *via* the AGP website or programmatically via the AGP API (https://github.com/ATCC-Bioinformatics/genome_portal_api). This REST-API will allow researchers to find specific genome assemblies and associated metadata as well as download assemblies for specific organisms. Full documentation of the methods and protocols used for data production for the AGP can be found online as well (https://docs.onecodex.com/en/collections/2314023-the-atcc-genome-portal). It meets the requirements of a FAIR data resource (14) and is listed with FAIRsharing.org (https://doi.org/10.25504/FAIRsharing.154974), SciCrunch.org (RRID: SCR_021330), and R3data.org (http://doi.org/10.17616/R31NJN33).

## Data Availability

Access channels for the data include downloads via the AGP website or programmatically via the AGP API (https://github.com/ATCC-Bioinformatics/genome_portal_api).
